# Serum uric acid as a potential marker for heart failure risk in men on antihypertensive treatment: The British Regional Heart Study

**DOI:** 10.1016/j.ijcard.2017.11.083

**Published:** 2018-02-01

**Authors:** S. Goya Wannamethee, Olia Papacosta, Lucy Lennon, Peter H. Whincup

**Affiliations:** a UCL Department of Primary Care & Population Health, UCL Medical School, Rowland Hill Street, London, NW3 2PF, UK; b Population Health Research Institute, St George's, University of London, Cranmer Terrace, London, SW17 0RE, UK

**Keywords:** Serum uric acid, Heart failure, Epidemiology, Hypertension

## Abstract

The role of serum uric acid (SUA) as a prognostic marker for incident heart failure (HF) in hypertensive subjects is uncertain. We have prospectively examined the relationship between SUA and incident HF in 3440 men aged 60–79 years separately in those on and not on antihypertensive treatment who were followed up for a mean period of 15 years. Men on SUA lowering drugs and those with history of HF or myocardial infarction were excluded. There were 260 incident HF cases. The men were divided into three groups of SUA concentrations/levels (< 350, 350–410 and > 410 μmol/L). Raised SUA was associated with significantly increased risk of HF in men on antihypertensive treatment (*N* = 949) but not in those without (*N* = 2491) (*p* = 0.003 for interaction). In men on antihypertensive treatment those with hyperuricemia (> 410 μmol/L) had the most adverse biological risk profile for HF including the highest rates of atrial fibrillation and renal dysfunction and the highest mean level of BMI, c-reactive protein and cardiac function (cardiac troponin T). Treated hypertensive men with SUA levels > 410 μmol/L showed an increase in risk of HF of more than twofold compared to those on treatment with levels < 350 μmol/L even after adjustment for lifestyle characteristics and biological risk factors [adjusted hazard ratio 2.26 (1.23,4.15)]. SUA improved prediction of HF beyond routine conventional risk factors (*p* = 0.02 for improvement in c-statistics). SUA as a marker of increased xanthine oxidase activity may be a useful prognostic marker for HF risk in older men on antihypertensive treatment.

## Highlights

• Raised serum uric acid (SUA) is associated with increased risk of heart failure in older men on antihypertensive treatment
• Treated hypertensive men with raised SUA have the most adverse risk profile for heart failure including underlying ischaemia
• Monitoring of SUA in older hypertensive patients may identify high risk patients who would benefit from further investigation

## 1 Introduction

Serum uric acid (SUA) is the end product of purine metabolism in humans; hyperuricaemia is commonly found in patients with heart failure (HF) and hypertension [1], [2]. The association of SUA and coronary heart disease has long been recognised [1] and has sparked enormous debate about the role of SUA as a risk factor for CHD and the treatment of hyperuricemia especially in hypertensive patients [1], [3], [4], [5]. In more recent years several studies and meta-analysis have reported raised SUA to be associated with increased risk of incident HF in population studies [6], [7], [8], [9], [10]. Whether this association is causal is still a matter of debate. However a recent Mendelian randomisation study provided no evidence that the association between SUA and HF is causal, suggesting that SUA may be only a risk marker rather than a causal factor in the development of HF [11]. Hypertension is a major risk factor for HF and regardless of whether SUA is causally related to HF, SUA may be a useful biomarker of increased HF risk in hypertension [12]. Recent studies have shown a strong association between SUA levels and AF [13], a major risk factor for HF and one study has shown SUA to predict HF in those with hypertension [14]. SUA is dependent on xanthine oxidase (XO) activity, a known cause of oxidative stress [15] which is implicated in the pathophysiology of HF [15], [16] as well as hypertension [17]. SUA may be a marker of increased XO activity which is up-regulated in the failing heart [15] and may thus identify patients with increased HF risk. SUA concentration is commonly measured in hypertensive patients and although a number of studies have shown SUA to be an independent marker of CVD risk in hypertensive subjects [18], [19], there is limited prospective data regarding the association between raised SUA and HF, especially in the older hypertensive adult population, who are at particularly high risk of developing HF. Whether SUA add to the prediction of HF beyond routine markers in older hypertensive patients has seldom been assessed. We have therefore examined the association between raised SUA and incident HF in older men who are on antihypertensive treatment, as well as in those who are not on antihypertensive treatment.

## 2 Subjects and methods

The British Regional Heart Study is a prospective study involving 7735 men aged 40–59 years drawn from one general practice in each of 24 British towns, who were screened between 1978 and 1980 [20]. The population studied was socio-economically representative and comprised predominantly white Europeans (> 99%). In 1998–2000, all surviving men, then aged 60–79 years, were invited for a 20th year follow-up examination, on which the current analyses are based. Ethical approval was obtained from all relevant local research ethics committees. All men completed a mailed questionnaire providing information on their lifestyle and medical history, had a physical examination and provided a fasting blood sample. The samples were frozen and stored at − 20 °C on the day of the collection and transferred in batches for storage at − 70 °C until analysis, carried out after no more than one freeze-thaw cycle. 12 lead electrocardiograms were recorded using a Siemens Sicard 460 instrument and were analyzed using Minnesota Coding definitions at the University of Glasgow ECG core laboratory. Men were asked whether a doctor had ever told them that they had angina or MI, HF or stroke; details of their medications were recorded at the examination including use of blood pressure lowering drugs (BNF code 3.1) and non-steroidal anti-inflammatory drugs (NSAIDs) (BNF code 10.1.1). 4252 men (77% of available survivors) attended for examination. 4034 men had blood measurement of SUA. We excluded men with prior HF or myocardial infarction (MI) (*N* = 508) in the examination and a further 86 men on treatment for gout (SUA lowering drugs) leaving 3440 men for analysis.

### 2.1 Cardiovascular risk factor measurements at 1998–2000

Anthropometric measurements including body weight and height were carried out. Details of measurement and classification methods for smoking status, physical activity, social class, alcohol intake, blood pressure and blood lipids in this cohort have been described [21], [22]. SUA was measured with an enzymatic colorimetric assay using a Hitachi 747 automated analyser. C-reactive protein (CRP) (marker of inflammation) was assayed by ultra sensitive nephelometry (Dade Behring, Milton Keynes, UK). Predicted glomerular filtration rate (eGFR) (measure of renal function) was estimated from serum creatinine using the equation eGFR = 186 × (Creatinine / 88.4)^− 1.154^ × (Age)^− 0.203^ [23]. N-terminal pro-brain natriuretic peptide (NT-proBNP) was determined using the Elecsys 2010 (Roche Diagnostics, Burgess Hill, UK) [22]. Troponin T was measured by a high sensitivity method on an e411 (Roche Diagnostics, Burgess Hill, UK) using the manufacturers calibrators and quality control material. The low control CV was 6.6%, and high control CV 3.0%, and the assay limit of detection was 3 pg/ml. Electrocardiographic left ventricular hypertrophy (LVH) was defined according to the relevant Minnesota codes (codes 3.1 or 3.3). Atrial fibrillation (AF) was defined according to the Minnesota codes 8.3.1 and 8.3.3.

### 2.2 Follow-up

All men have been followed up from initial examination (1978–1980) for cardiovascular morbidity [20] and follow-up has been achieved for 99% of the cohort. In the present analyses, all-cause mortality and morbidity events are based on follow-up from re-screening in 1998–2000 at mean age 60–79 years to June 2014, a mean follow-up period of 15 years (range 14–16 years). Survival times were censored at date of HF, death from any cause or end of the study follow-up period (June 2014) whichever occurred first. Evidence of non-fatal MI and HF was obtained by ad hoc reports from general practitioners supplemented by biennial reviews of the patients' practice records (including hospital and clinic correspondence) to the end of the study period. Incident non-fatal HF was based on a confirmed doctor diagnosis of HF from primary care records and confirmed by a review of available clinical information from primary and secondary care records (including symptoms, signs, investigations, treatment response) to ensure that the diagnosis was consistent with the current recommendations on HF diagnosis [24]. The incidence and determinants of HF cases identified using this process have already been reported and are consistent with the results from other studies [21], [22]. Incident HF included incident non-fatal HF as well as death from HF (ICD 9th revision code 428 or ICD10th revision I28).

### 2.3 Statistical methods

The men were divided into three groups based on the tertile distribution of SUA in all men (< 350, 350–410 and > 410 μmol/L). Hyperuricemia in men is generally defined as those with SUA > 420 μmol/L [25]. Thus those in the upper tertile were effectively hyperuricaemic. Kaplan–Meier methods were used to calculate the cumulative HF incidence for the three groups of SUA; the log-rank test was used to evaluate differences in the HF rates for these groups. Analyses were carried out stratified by antihypertensive treatment status. Cox proportional hazards model was used to assess the multivariate-adjusted hazards ratio (HR) in a comparison of the three SUA groups as well as in a 1 SD increase in SUA. The proportional hazard assumption was examined using time-varying covariates, calculating interactions of predictor variables and a function of survival time and including them in the models. Examination of time-varying covariates indicated no violation of the proportionality assumption. The distributions of NT-proBNP, cTnT and CRP were skewed and log transformation was used to normalise these factors. Receiver-operating characteristic (ROC) curves and areas under the curve (AUC) (c-statistics) were used to assess the ability of SUA to predict HF in men on antihypertensive treatment and who had no history of HF or MI beyond a score which included conventional routine risk factors as well as how SUA predicted beyond the Health ABC HF score. Conventional routine risk factors included established risk factors for HF routinely obtained in clinical practice e.g. age obesity, hypertension, history of diabetes and history of angina. The Health ABC HF score includes age, smoking, eGFR, heart rate, left ventricular hypertrophy, albumin, systolic blood pressure, history of angina, fasting blood glucose and antihypertensive treatment [26]. To assess whether the association between SUA and incident HF may be due to the development of incident CHD which in turn results in increased risk of HF, we adjusted for incident CHD by fitting CHD as a time dependent covariate.

## 3 Results

During the mean follow-up period of 15 years there were 260 incident HF events (6.31/1000 person years), in the 3440 men without MI or HF and who were not on SUA lowering drugs.

**Fig. 1:**
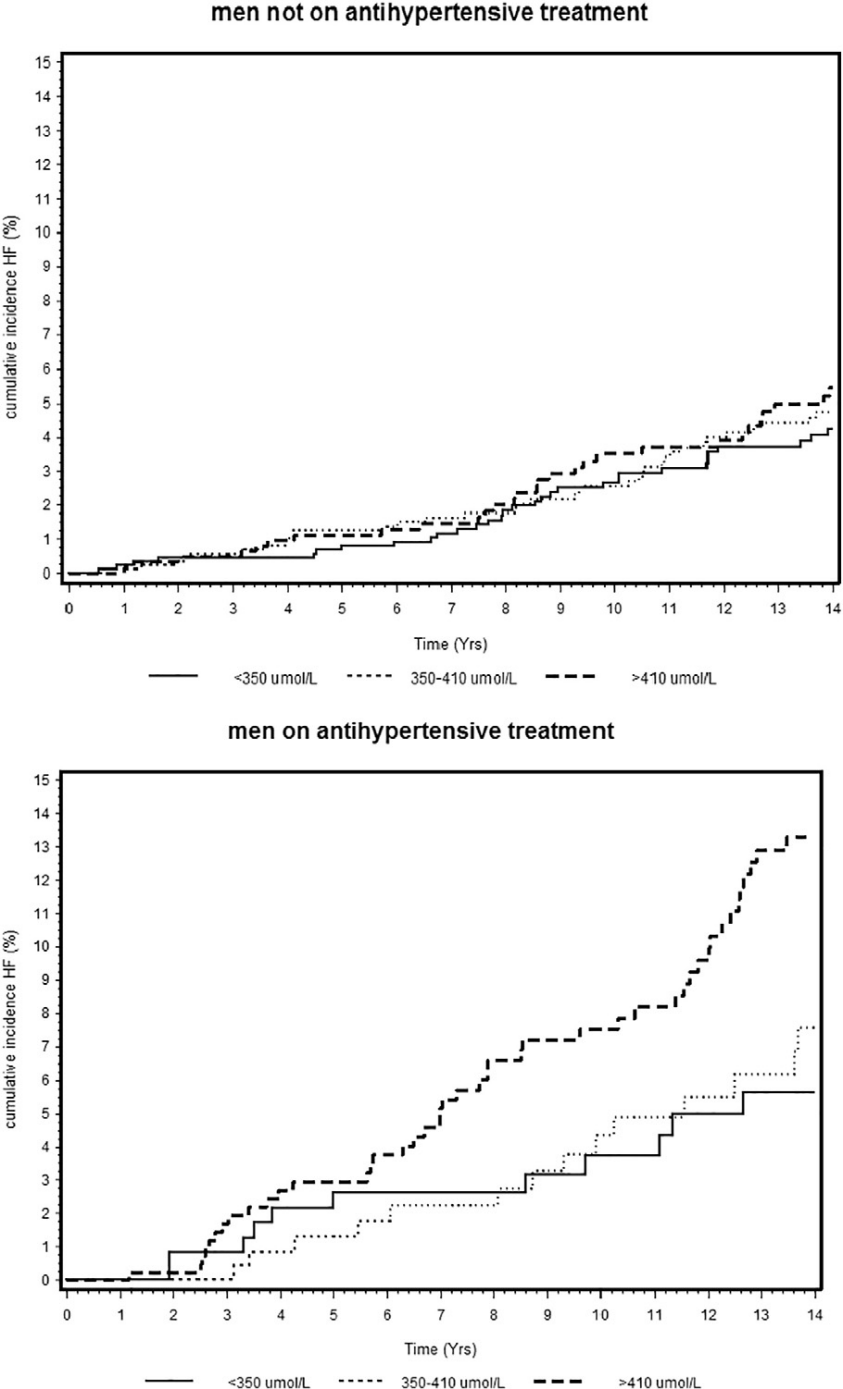
Kaplan-Meier curve of cumulative heart failure incidence by tertiles of SUA in men on antihypertensive treatment and in men not on antihypertensive treatment. Log rank test: Men not on antihypertensive treatment *p* = 0.57; Men on antihypertensive treatment *p* < 0.001;

Fig. 1 show the Kaplan–Meier estimates of the cumulative incidence of HF by tertiles of SUA separately by antihypertensive treatment status. Risk of HF was increased only in those in the top tertile of the SUA distribution, and this was only seen in men on antihypertensive treatment. No association was seen between SUA and HF in those not on treatment and a test for interaction confirmed a significant difference in the relation between SUA and incident HF in those on and not on antihypertensive treatment (*p* = 0.02). We therefore present the findings stratified by antihypertensive medication status.

Table 1 shows the association between SUA and biological risk factors in men on and not on antihypertensive treatment. With the exception of blood pressure, the patterns of association between SUA and biological risk factors were similar in both men on and not on treatment. Men on antihypertensive treatment with raised SUA had the highest levels of cardiovascular risk factors including renal dysfunction, AF, systolic blood pressure, cTnT and the lowest level of HDL-C and FEV_1_.

**Table 1:** SUA and biological risk factors in men on and not on antihypertensive treatment.

	Men not on treatment	> 410	*p*-Difference	Men on treatment	> 410	*p*-Difference
	SUA (μmol/L)			SUA(μmol/L)		
	< 410			< 410		
No of men	1871	668		485	418	

*Type of BP treatment*						
% BB				41.4	39.2	0.50
% diuretics				20.2	31.6	< 0.0001
% ACEi				16.5	34.2	< 0.0001
Age	68.2 (5.5)	68.1 (5.4)	0.86	69.4 (5.5)	69.7 (5.2)	
%AF	1.7	3.0	0.03	4.7	7.7	0.07
% renal dysfunction	8.2	20.1		15.7	28.7	
% LVH	6.5	7.5	0.37	10.1	10.8	0.75
BMI kg/m^2^)	26.1 (3.4)	27.5 (3.5)	< 0.0001	27.0 (3.5)	28.4 (3.8)	< 0.0001
SBP (mmHg)	147.0 (23.7)	150.4 (22.4)	< 0.0001	155.8 (25.0)	156.6 (23.4)	0.62
HDL-C	1.38 (0.35)	1.29 (0.34)	< 0.0001	1.32 (0.35)	1.23 (0.30)	< 0.0001
Log HOMA-IR	0.66 (0.71)	0.84 (0.66)	< 0.0001	0.88 (0.84)	1.04 (0.70)	0.001
FEV1 (L)	2.68 (0.68)	2.62 (0.63)		2.50 (0.61)	2.48 (0.62)	0.63
CRP (mg/L)⁎	1.46 (0.67–2.95)	1.77 (0.90–3.60)	< 0.0001	1.99 (0.95–3.93)	2.29 (1.16–4.66)	0.06
Heart rate (b/min)	65.60 (11.4)	67.73 (13.2)	< 0.0001	64.9 (14.2)	64.7 (14.95)	0.81
NT-proBNP (pg/ml)⁎	76.7 (40–141)	76.7 (36–147)	0.93	127.7 (64–269)	144.0 (61–275)	0.74
cTnT (pg/ml)⁎	11.0 (8.2–14.8)	12.1 (8.9–15.9)	< 0.0001	11.8 (9.0–15.7)	13.5 (9.7–17.7)	0.0002

⁎ Geometric mean (IQ range).

In men not on antihypertensive treatment, no association was seen between SUA and HF (Table 2). When this group was separated into those with measured hypertension (systolic blood pressure ≥ 160 or DBP ≥ 90) and those without, no association was seen in either group (data not shown). In contrast, in men on antihypertensive treatment, hyperuricemia (> 410 mmol/L) was significantly associated with increased risk of HF even after adjustment for a wide range of HF risk factors including AF, BMI, HDL-C, systolic blood pressure, renal dysfunction, CRP and cardiac function (NT-proBNP and cTnT). Further adjustment for incident CHD made little difference to the findings. The increased risk of HF associated with hyperuricaemia remained even after exclusion of men on diuretics [adjusted HR = 1.97 (1.01,3.81);model 4] or exclusion of men with renal dysfunction [adjusted HR = 2.56 (1.30,5.02);model 4] or the exclusion of 84 men on NSAIDs [adjusted HR = 2.37 (1.29,4.35); model 4].

**Table 2:** Rates/1000 person-years and adjusted hazard ratios (HR) (95%CI) for incident HF by serum uric acid levels in men with no prevalent MI or HF by antihypertensive status.

		Serum uric acid (μmol/L)			
	< 350	350–410	> 410	Increase in 10 μmol/L	*p*-Trend
		All men			
No of men	1186	1170	1084		
per-yrs (n)	4.8 (67)	5.8 (82)	8.6 (111)		
		Not on antihypertensive treatment			
No of men	948	923	668		
Rate/1000 per-yrs (n)	4.5 (50)	5.4 (61)	5.4 (43)		
Age-adjusted HR	1.00	1.16 (0.80,1.69)	1.23 (0.83,1.83)	1.013 (0.991,1.035)	0.25
Model 1	1.00	1.24 (0.84,1.83)	1.11 (0.73,1.70)	1.005 (0.983,1.028)	0.65
		On antihypertensive treatment			
No of men	238	247	418		
Rate/1000 per-yrs. (n)	5.6 (15)	7.2 (20)	13.6 (63)		
Age-adjusted HR	1.00	1.30 (0.66,2.56)	2.39 (1.36,4.21)	1.033 (1.011,1.056)	0.003
Model 1	1.00	1.44 (0.72,2.90)	2.59 (1.42,4.75)	1.035 (1.010,1.061)	0.006
Model 2	1.00	1.44 (0.71,2.90)	2.49 (1.36,4.56)	1.035 (1.011,1.061)	0.004
Model 3	1.00	1.54 (0.76,3.13)	2.42 (1.29,4.54)	1.028 (1.002,1.054)	0.02
Model 4	1.00	1.43 (0.71,2.89)	2.26 (1.23,4.15)	1.027 (1.001,1.050)	0.04

Model 1 adjusted for age, smoking, social class, alcohol intake, physical activity, BMI, HDL-C, diabetes, SBP, prevalent stroke, prevalent angina.

Model 2 = Model 1 + AF + renal dysfunction + CRP.

Model 3 = Model 2 + NT-proBNP.

Model 4 = model 2 + cTnT.

The elevated risk associated with hyperuricaemia was seen in those with controlled blood pressure (defined as systolic blood pressure < 150 and diastolic blood pressure < 90;*N* = 321) and those with uncontrolled blood pressure (*N* = 585) although the association was somewhat stronger in those with controlled hypertension compared to those with uncontrolled blood pressure (adjusted HR = 4.70 (1.53,14.44) vs 2.09 (0.98,4.48);model 4]. However a test for interaction was not significant (*p* = 0.47).

Table 3 shows the c-statistics for the conventional risk score and the Health ABC HF score and the improvement in C-statistics in models with and without SUA. SUA added significantly to HF prediction beyond that provided by either risk score. However, a model which included conventional risk factors and NT-proBNP showed no significant improvement on the addition of SUA [c statistic = 0.676 (0.624–0.728) versus 0.691 (0.640–0.742) with SUA]; evidence for improvement *p* = 0.14].

**Table 3:** Improvement in c statistics for conventional models and the ABC health score models with and without NT-proBNP and SUA in men on antihypertensive treatment and with no diagnosed MI or HF.

Model	c-statistics	*p*-Value improvement
Conventional risk factors	0.621 (0.567,0.675)	–
Conventional risk factors + SUA	0.654 (0.601,0.707)	*p* = 0.02
ABC score	0.617 (0.565,0.671)	–
ABC score + SUA	0.649 (0.597,0.701)	*p* = 0.02
Conventional risk factors + NT-proBNP	0.676 (0.624,0.728)	–
Conventional risk factors + NT-proBNP + SUA	0.691 (0.640,0.742)	*p* = 0.14

Conventional risk factors (routine clinical risk factors) include age, BMI, systolic blood pressure, renal function, history of diabetes, stroke and angina.

ABC score include age, smoking, eGFR, heart rate, left ventricular hypertrophy, albumin, systolic blood pressure, history of angina and fasting blood glucose.

SUA fitted as tertiles in the model.

## 4 Discussion

In this study of older men with no history of an MI or HF raised SUA was associated with significantly increased risk of HF which was only seen in those who were on antihypertensive treatment. No association was seen between SUA and HF risk in those who were not on antihypertensive treatment. Our finding extends those of previous studies of SUA and HF by examining the relationship by antihypertensive medication status and by examining the prognostic value of SUA and improvement in risk prediction in those with hypertension. The increased risk of HF in those on treatment was seen even after adjustment for a wide range of HF risk factors including renal dysfunction, inflammation and cardiac function (NT-proBNP and cTnT). In a recent meta-analysis which included 17 studies SUA has shown to be an independent predictor of CVD morbidity and mortality in hypertensive patients [19] and our findings extend this to HF outcome.

### 4.1 SUA and incident HF

A number of prospective studies and meta-analysis have shown SUA to predict HF in the general population [6], [7], [8], [9], [10]. However, few studies have examined the relationship by antihypertensive treatment status. We have shown SUA to be associated with increased risk of HF in the general population but this was only seen in those on treatment. Although numerous studies have examined the association between SUA and risk of CHD events and CVD mortality in hypertensive patients few studies have reported on the specific effect of SUA on HF risk in hypertensive patients. The Primary Preventive Trial in Goteborg showed that SUA predicted HF in treated hypertensives [14]. However, in the Cardiovascular Health Study SUA did not predict HF in those with hypertension or those on antihypertensive treatment [10]. The reason for this discrepancy is uncertain but it may relate to the use of SUA lowering drugs in hypertensive subjects as these patients were not excluded and many may have been on SUA lowering drugs which may affect the true association of hyperuricemia with HF if those on SUA lowering drugs were included in the normal SUA group. We have shown that SUA improves prediction in HF risk beyond routine clinical risk factors for HF including BMI, hypertension, renal dysfunction pre-existing diabetes, stroke and angina. SUA also improved prediction beyond that provided by the ABC heart failure score. Although SUA did not improve HF risk prediction beyond NT-proBNP, SUA is a routine marker and easily measured in primary care and may help identify high risk subjects who would benefit from further evaluation of cardiac dysfunction and AF.

The findings that SUA predicts HF in those on treatment only suggests that SUA does not have an intrinsic relationship with HF but may be a marker of other pathways.

Raised SUA could also be due to impaired renal clearance and increased production of urate and renal dysfunction has been associated with HF risk. We have shown a strong association between renal dysfunction and SUA but the increased risk of SUA and HF was seen even after exclusion of men on diuretics or men with renal dysfunction. SUA has been associated with increased risk of developing AF [13] and SUA was associated with prevalent AF but adjustment for AF did not alter the findings. We did not have information on AF during follow-up and it is possible that part of the association may be mediated by the development of AF. Elevated serum SUA could be a marker of underlying tissue ischaemia [27]. The finding that SUA was associated with cTNT suggests that it may be detecting men with sub-clinical cardiac dysfunction. However although part of the association was mediated by cTnT, the association remained after further adjustment for cTnT. We observed no association between SUA and NT-proBNP a marker of ventricular stress. Thus the increase risk of HF in hyperuricemic men compared to normouricemia men is unlikely to be due to the presence of asymptomatic HF.

### 4.2 SUA and xanthine oxidase activity

Evidence from a number of studies suggests that hyperuricaemia is associated with HF when it is a marker of increased xanthine oxidase activity [9], [10]. The finding that SUA predicted HF in hypertensive treated patient only is consistent with this notion. SUA is produced from the metabolism of purines by XO. XO activity has been shown to play an important role in the pathogenesis of hypertension [17] and is also involved in the production of reactive oxygen species (ROS). Experimental and animal models suggest that oxidative stress which is characterised by excessive production of reactive oxygen species (ROS) and reduction of antioxidant defence capacity may play an important role in the pathophysiology of HF [28]. Hyperuricemia in the presence of essential hypertension may be a compensatory response to counteract excessive oxidative stress and thus represent a marker of increased XO expression activity and oxidative stress.

### 4.3 Lowering of SUA and CVD

There has been much debate on the potential benefits of lowering SUA levels and in recent years much attention has focused on the potential benefits of XO inhibitors [4]. A recent Cochrane review concluded that there is insufficient evidence for use of allopurinol an XO inhibitor, or other uric acid lowering drug as the initial treatment of hypertension [5]. However, most trials have focused on CVD or CHD as the end point and few have examined HF specifically. A recent observational study in gout patients showed no reduction in risk of HF in those on XO inhibitors compared to untreated hyperuricaemia [29]. However, the benefits of XO may only be apparent in selected high risk patients and in those with increased oxidative stress such as in older hypertensive subjects in whom hyperuricemia may be a marker of XO activity. Trials in subjects such as diabetes and hypertensive patients have shown beneficial effects of allopurinol on endothelial dysfunction, left ventricular hypertrophy and arrhythmias [30], major risk factors for HF. However, whether the use of XO inhibitors in hypertensive patients reduces incident HF is yet to be established.

### 4.4 Strengths and limitations

The strength of this study is as a representative cohort with a wide range of HF risk factors measured and high follow up rates. However, it was based on an older, predominantly white, male population of European origin, so that the results cannot be generalized directly to women, or to younger populations or other ethnic groups. The current findings are based on doctor diagnosed HF, which is likely to underestimate the true incidence of HF in this study population. However, the other risk factor associations to HF risk in this report and in our previous report on obesity, NT-proBNP and lung function and HF [21], [22], [31] generally accord with prior data and therefore suggest potential external validity for our findings. Information on echocardiogram measurements was not available and we were not therefore able to differentiate systolic and diastolic HF. SUA was only measured at a single point in time, so that strengths of associations may be underestimated.

### 4.5 Clinical implications and conclusion

In this study we have shown SUA to be associated with increased risk of HF in older men on antihypertensive treatment which is independent of known risk factors. The findings that hypertensive men with hyperuricaemia had the most adverse risk profile for HF (including underlying ischaemia, atrial fibrillation, inflammation), raises the issue of whether SUA levels should be routinely monitored in older hypertensive patients in primary care. Direct measurement of XO is difficult; SUA is easy to measure and may be a marker of XO activity in hypertension. Current guide lines in the National Institute for Health and Care Excellence (NICE) do not include SUA in the list of risk factors review for cardiovascular disease. Measurement of SUA in older hypertensive patients in primary care may help identify high risk patients who may benefit from further evaluation of subclinical cardiac dysfunction and pharmacological intervention. Primary intervention trials in older hypertensive people at high risk of HF are needed to confirm whether XO inhibitors would reduce risk of HF in this group.

## Authors' contribution

SGW initiated the concept and design of the paper, analysed the data and drafted the manuscript. PHW contributed to the interpretation of the data. OP contributed to the analysis of the paper. PHW and LL contributed to the acquisition of the data. All authors revised it critically for important intellectual content and approved the final version of the manuscript. SGW is the guarantor for the manuscript and has full access to all the data in the study and takes responsibility for the integrity of the data and the accuracy of the data analysis.

## Conflict of interest

The authors report no relationships that could be construed as a conflict of interest.

## Disclosure

None.

## Funding

The British Regional Heart Study is supported by a British Heart Foundation (BHF) Programme grant (RG/13/16/30528).
